# Pervasive, hard-wired and male: Qualitative study of how UK adolescents view alcohol-related aggression

**DOI:** 10.1371/journal.pone.0191269

**Published:** 2018-02-06

**Authors:** Lydia Whitaker, Stephen L. Brown, Bridget Young, Richard Fereday, Sarah M. Coyne, Pamela Qualter

**Affiliations:** 1 Department of Psychological Sciences, University of Liverpool, Liverpool, United Kingdom; 2 College of Family, Home and Social Sciences, Brigham Young University, Provo, Utah, United States of America; 3 Institute of Education, University of Manchester, Manchester, United Kingdom; Harvard Medical School, UNITED STATES

## Abstract

Laboratory studies of alcohol-inexperienced adolescents show that aggression can be primed by alcohol-related stimuli, suggesting that alcohol-related aggression is partly socially learned. Script theory proposes that alcohol-related aggression ‘scripts’ for social behaviors are culturally-available and learned by individuals. The purpose of the study was to understand the content and origins of alcohol-related aggression scripts learned by adolescents. This qualitative focus group study of 40 adolescents (ages 14–16 years) examined alcohol-related aggression scripts. Participants believed aggression and severe injury to be pervasive when young people drink. Viewed through a biological lens, participants described aggression as an *‘instinctive’* and *‘hard-wired*’ male trait facilitated by intoxication. As such, alcohol-related aggression was not seen as intended or personally controllable and participants did not see it in moral terms. Females were largely viewed as either bystanders of inter-male aggression or potential victims of male sexual aggression. Participants attributed their views on the frequency and nature of alcohol-related aggression to current affairs and reality television, which they felt portrayed a reality of which they had little experience. The origins of the explicitly biological frameworks that participants used seemed to lie in pre-existing beliefs about the nature of gender differences. Perceptions of the pervasiveness of male alcohol-related aggression, and the consequent failure to view alcohol-related aggression in moral terms, could dispose some young people to alcohol-related aggression. Interventions could target (1) the beliefs that alcohol-related aggression is pervasive and uncontrollable in males, and (2) participants’ dysfunctional views of masculinity that underpin those beliefs.

## Introduction

Interpersonal violence causes morbidity and mortality in young people [1], and is strongly associated with nighttime weekend drinking in pubs, bars and clubs [2, 3]. Laboratory experiments suggest convincing pharmacological links between consumption of alcohol and aggression [4]. Non-alcohol placebo drinks also induce aggressive responses, which suggests that aggression and alcohol are linked through beliefs and expectations concerning alcohol and aggression [5].

Researchers have documented widely shared beliefs and expectations concerning the permissibility and performance of alcohol-related aggression amongst drinkers [6, 7, 8]. Largely, although not exclusively confined to males (e.g., [9]), aggression is described by drinkers as (1) a source of stimulation, and (2) a way of enhancing and protecting and social status through capability in physical and verbal conflict [10, 6]. For example, in the UK, ‘lad’ culture ties together alcohol and aggression within a rubric of distorted but powerful masculine values emphasizing status, hardness, and thrill-seeking.

Researchers have shown less interest in how individuals acquire these beliefs and expectations. One possibility is that children and adolescents learn to associate alcohol with aggression before they start drinking. Evidence from adolescents below the legal drinking age supports that idea. Brown et al. [11] showed that presentations of images of alcoholic drinks led to greater laboratory aggression than similar images of non-alcoholic beverages. As most of their participants did not drink, and alcohol use was statistically controlled in those who did, this finding eliminates experiential learning explanations. It suggests that alcohol-related aggression is partly socially learned, and that learning predates drinking. In the current study, we use the concept of a social script to investigate the nature of adolescents’ understandings of alcohol-related aggression, how they developed these understandings and possible influences on their behavior.

### Social scripts and alcohol-related aggression

There is little literature on social learning of alcohol-related aggression in young people, but researchers have extensively explored acculturation of young people to alcohol use [12, 13] and non-alcohol aggression [14]. A key theoretical construct is that of a social script—a complex and sequentially organized representation of expected and appropriate social behavior that is shared within social or cultural groups and learned by individuals through their social and cultural interactions. Scripts encompass 1) a social context that elicits the script, 2) a sequence of accepted and permissible actions, and 3) the consequences of those actions [15].

Qualitative studies describe complex and widely understood alcohol aggression scripts [10, 6, 16]. Aggression is seen, or justified, as an assertion of heterosexual male working class identities [10, 8]. Ritualized rules of engagement and disengagement define the permissibility of aggression, appropriate responses to provocation, and social and personal rewards such as excitement and reputational enhancement. Alcohol is seen to serve two purposes: (1) a desirable means of intoxication, and (2) mitigation for engagement in aggression or other anti-social behavior [6].

Yet, the content of alcohol-related aggression scripts also reflects negative personal, social, and moral implications of alcohol-related aggression [7]. Thus, scripts contain parameters that proscribe contextually inappropriate aggression, endorse de-escalation and disengagement techniques that permit honorable conflict resolution, and promote intervention to stop aggression by others [17, 7, 8]. For example, research has shown that less-experienced drinkers are more vulnerable to alcohol-related aggression, compared to older drinkers, because they lack a nuanced understanding of the script components that control violence [10, 6, 7, 8].

As reported alcohol-related aggression is perpetrated by males [2], researchers have focused on male accounts. Typically, women are afforded roles of spectator, mediator, or victim. However, the few studies of females, who engage in alcohol-related aggression against males or other females, show consistency with sub-cultural norms that are broadly similar to, and possibly influenced by, male norms [9].

Script theory posits that social knowledge is actively constructed by individuals from multiple sources of information to make sense of the demands of their particular sociocultural environments. Thus, we frame this research within a tradition of adolescent development research that emphasizes a constructive and critical participation in the learning of adult roles framed by developmental and sociocultural opportunity [18]. This is exemplified by research on young people’s understandings of alcohol use [12, 13]. Learning is conceptualized as a cumulative process of active enquiry that is influenced but not determined by social and cultural contexts, such as media, family and peers [19].

### The current study

If young people are disposed to alcohol-related aggression by scripts learned before they fully engage in drinking, preventive interventions could target nascent scripts by either disrupting their formation or challenging already-formed scripts [20]. Such interventions cannot be developed without understanding the content, origins, and potential behavioral implications of scripts. Thus, we examined script representations in male and female adolescents aged 14–16 years. Because this is the first such investigation, we did not formulate testable hypotheses, instead employing inductive qualitative methods. We used focus group interviews to promote interaction and discussion that can provide insights beyond those that could be accessed via individual interviews [21].

## Materials and method

We conducted seven focus groups with a total across groups of 40 adolescents [*M* age = 14 years and 7 months, 16 females] aged between 14 and 16 years (Table 1). We sampled for maximum variation [22] by selecting four secondary schools representing areas of high and low deprivation; urban, rural, and semi-rural areas; and high and low concentrations of adolescent (13–19 years) alcohol-related offenders living within local areas (Lancashire County Council’s youth offending geodata). Schools 1–3 were in higher deprivation semi-rural areas with higher concentrations of offenders in the local area (4.07–7.73 per 10,000), schools 4–7 were in low deprivation urban areas with low concentrations of offenders (1.07–4.06 per 10,000 adolescent residents). Sampling continued until data saturation [23]. Ethical approval was obtained from the University of Liverpool (RETH000731) and the University of Central Lancashire (PSYSOC207CA). Participants were fully informed of the aims and methods of the study and gave written consent. Parents/guardians also gave written consent. Ethical approval was granted on the basis that participants were discouraged from discussing either personal or family experiences of alcohol use of alcohol-related aggression. All 40 participants approached chose to participate.

**Table 1:** Demographic characteristics of participants (N = 40).

Focus Group number		Gender	Age	School number	Deprivation level	Urban/Rural Area	Offender concentration*
1				1	Low	Urban	4.07–7.73
	**Participant 1**	M	14				
	**Participant 2**	M	16				
	**Participant 3**	F	16				
	**Participant 4**	F	16				
	**Participant 5**	M	16				
2				1	Low	Urban	4.07–7.73
	**Participant 1**	F	14				
	**Participant 2**	F	14				
	**Participant 3**	F	14				
	**Participant 4**	F	14				
3				1	Low	Urban	4.07–7.73
	**Participant 1**	F	14				
	**Participant 2**	F	14				
	**Participant 3**	M	14				
	**Participant 4**	M	14				
	**Participant 5**	M	14				
	**Participant 6**	M	14				
	**Participant 7**	M	14				
4				4	High	Rural	1.07–4.06
	**Participant 1**	M	16				
	**Participant 2**	M	16				
	**Participant 3**	M	16				
	**Participant 4**	M	16				
	**Participant 5**	M	16				
	**Participant 6**	M	16				
5				3	High	Semi-rural	1.07–4.06
	**Participant 1**	M	15				
	**Participant 2**	M	15				
	**Participant 3**	M	15				
	**Participant 4**	M	15				
	**Participant 5**	M	15				
	**Participant 6**	M	15				
6				3	High	Semi-rural	1.07–4.06
	**Participant 1**	F	15				
	**Participant 2**	M	15				
	**Participant 3**	M	15				
	**Participant 4**	M	15				
	**Participant 5**	F	15				
	**Participant 6**	M	15				
7				2	Low	Semi-rural	1.07–4.06
	**Participant 1**	F	14				
	**Participant 2**	F	15				
	**Participant 3**	F	15				
	**Participant 4**	F	15				
	**Participant 5**	F	14				
	**Participant 6**	F	15				

* Rate per 1,000 of 13–19 year-olds arrested for an alcohol-related offence, October 2012-September 2013.

### Procedure

Each focus group comprised 4–7 participants. Each group was largely of the same age. Participants were approached by the school, and participant and parent consent obtained by the researchers. Discussions took place on school premises. To initiate dialogue participants were given two black and white photographs, one of a group of males and another of a group of females, drinking alcohol. Drinkers were clearly intoxicated and exuberant, but judged by the authors to be neither angry nor aggressive. Photographs of mixed gender groups were not used because mixed groups inhibit aggression [3]. Participants were asked to individually consider what might happen to those drinkers during the evening and write brief notes to inform their contributions to the discussion. Note making was facilitated by the following questions: 1) Who are the people in the photographs and what do they look like? 2) How well do you think they will get on with each other and other people? 3) How do you think they may feel and what may they be thinking? 4) What might happen to them?

### Procedure

Group discussions were chaired by LW (PhD in Psychology), were participant-led and conversational. A teacher was present but did not speak. Participants did not know the interviewer before the groups were conducted. An interview guide covered the following topics: how participants understood alcohol-related aggression and how it arises, what social contexts alcohol-related aggression occurs in and participants’ views about social acceptability, and their perceptions of individuals who engage in alcohol-related aggression. Where participants’ contributions diverged from the guide, but were relevant to the project aims, the facilitator used prompts and follow-up questions to clarify and further explore contributions. New questions and probes and modifications of existing material were developed to revisit prior content and test emerging ideas. All participants were encouraged to contribute, and the facilitator ensured that individuals did not dominate discussions. Discussions lasted between 35–50 minutes.

Each focus group was audio-recorded and transcribed. Groups ran for an average of 41 minutes. Transcriptions included interruption/over-lapping speech, and audible features of speech and sub-vocalization such as hesitations, partial words, actions/sounds, incoherent speech, and intonation. Participants did not have the opportunity to comment on findings.

### Data analysis

Data analysis was informed by the constant comparative method [24], but sat outside of the constructivist philosophy associated with Grounded Theory. Rather, our philosophical orientation corresponded to subtle realism and pragmatism [25]. We conducted the analysis contemporaneously with the interviews, to develop interview questions to ‘test’ ideas raised from earlier interviews, continually comparing the data and the ongoing analysis to develop our interpretations. These themes were compared within and between transcripts to examine their fit to the data. LW began the analysis, reading each transcript several times and meeting periodically with SB and BY, who also read transcripts, to discuss key themes and categories, and to ‘test’ and refine the developing analysis. Divergences in the initial interpretations were identified and resolved. This led to the development of a preliminary coding framework, which LW used to code the transcripts. These reports, containing extensive data extracts, were circulated to the wider study team. This enabled investigator triangulation to scrutinise and refine the analysis. The analysis was iterative and cycled multiple times as earlier transcripts were revisited and sometimes reinterpreted in the light of insights from later transcripts. We recorded the development of conceptual themes during analysis using Microsoft Word [26].

Analysis focused on overt content, although we were mindful of the possibility that participant accounts might be influenced by self-presentation motives or merely flawed recall. Therefore, we also attended to participants’ use of language in expressing their views and to contradictions and inconsistencies in the accounts that could indicate hidden meanings. In particular, we were alert to the possibility that the group social context may create a false consensus. Thus, we monitored consistency of views and agreements and disagreements within and across groups. Quotes are accompanied by a code to indicate the focus group session number (e.g. *G1*), identification number (e.g. *P2*), and gender and age for each participant. Ellipses mark text or speech continuation that has been omitted for clarity and succinctness.

## Results

Males and females participated equally in the groups and expressed similar views. Alcohol-related aggression was seen as an almost exclusively male activity. While sexual aggression was occasionally mentioned, participants focused on male to male physical aggression. We developed three major themes from participants’ responses: 1) aggression is an inherent and inevitable consequence of innate male aggression, elicited through intoxication; 2) alcohol-related aggression as an (almost) excusable action; and 3) participants actively constructed their views of the frequency and forms of alcohol-related aggression from what they had seen in news and reality television. In the analysis, we were alert to how age, sex, deprivation, urban/rural location, and offender concentration differences may have influenced accounts. Groups 5–7 provided slightly longer and more detailed accounts but otherwise the accounts of the groups did not differ appreciably in content.

### Aggression is in males’ ‘genes’ which is elicited through intoxication and drinking context

Although not directed to think about alcohol-related aggression, participants saw physical aggression as an unremarkable and ubiquitous consequence of a normal night out for men: “*just a normal night out and I think it could get aggressive cos anything can happen really (G6; P3*, *M*, *15 yrs*.*)*. Accounts of the types of aggression that may occur involved unarmed fights, the use of glass as a weapon, and stabbings. Disability and death of males and sexual assault of females were described as possible outcomes of alcohol-related aggression. Initially, we felt that participants may have sought to exaggerate or dramatize their contributions, for enhancement within their peer group, or to provide interesting stories to satisfy the researcher. However, close inspection of participants’ accounts showed that accounts were detailed and largely coherent, and consistent within and across participants; providing some confidence that participants’ accounts reflected commonly-shared representations of alcohol-related aggression [27]. This can be particularly seen in their descriptions of how drinkers’ generally positive moods could quickly escalate into aggression after minor accidents or conflicts. The following examples are typically cogent;

“well they’ re just think they wanna have a laugh, but then if someone gets in the way then it starts to get a bit more personal maybe if people start gettin' in the way insults are thrown at each other and it just builds up into a fight and more and more people get involved so they’re not really thinking they’re just cos they’re drunk they’re like they’re not sensible or they’re not thinking properly as if they would when they are sober” (G6; P6; M 15yrs.). “Somebody might call em fat …cos they’ re like drunk they don’t know how to take it properly so they just take it in a really wrong way and they be like arr lets have a fight” (G3; P7, M,14 yrs.). “They feel happy while they’re drinking but then when they get too drunk they start falling out” (G2; P2, F, 14 yrs.). “Getting drunk, I think they will get on well with each other but might fight each other later on. Feel confident and they’re thinking everything will be all-right, but they might have too much to drink and everything will go violent (G1; P1, M, 14 yrs).”

Concerned about the possibility of false consensus, we monitored the extent to which participants agreed and disagreed with each other within groups. We noted frequent disagreements about how aggression may play out, suggesting that participants had not specifically tried to achieve consensus. The following exchange is from group 2; *“they might throw insults at each other and girls don’t like insults I guess (P1*, *F) probably tell like their secrets they’ve never told anyone (P2*, *F) I don’t think they fall out if its just a couple of people like I think you- you’ re right but if there’s more people then more agro (P4*, *F) yeah (P2*, *F)*.*”*

Participants of both genders saw males to be innately aggressive even when sober. They used a biological framework to explain male aggression, invoking genetic metaphors such as *‘instinctive’*, *‘hard-wired’* or *“in their genes” (G2; P1*, *F*, *14 yrs*.*)*. This idea of a genetic basis for male aggression was common and analogies to animals were frequently made; *“it’s hard wired in ya*…*cos you think of monkeys doing it* [aggression] *and like*… *group of lads going out to have a bit of fun and get drunk if like another group annoys them they could be prone to have a fight with them” (G6; P5*, *F*, *15 yrs*.*)*. “*guys can be more hostile… showing off like a peacock shows it’s feather off it’s like oo look at me (G5; P6*, *M*, *15 yrs*.*) (interrupts) Well it’s like stags”* (*G5; P2*, *M*, *15 yrs*.*)*. The use of genetic metaphors and animal analogies dehumanizes young males, portraying them as non-agentic and doomed to aggression by their very nature. Participants saw male dominance and competitiveness as particularly likely to contribute toward aggression, and spoke of how males sought to establish higher status relative to other males as a way to appeal to females; “*they’re marking their territory*, *you see two guys fighting over a women*…*you see animals fighting to get the women it’s like that with us we’re fighting to get the woman*…*prove their dominance to the woman” (G6; P6*, *M*, *15 yrs*.*)*. It was notable that no participant mentioned that males were innately aggressive or sexually coercive toward females.

Whilst males were seen to be aggressive, intoxicated or sober, alcohol was perceived to exacerbate these tendencies. Participants drew on the idea that alcohol disinhibits latent male aggression by creating feelings of being “*invincible”* and impairing normal judgment: “*you don’t think of the consequences (G6; P6*, *M*, *15 yrs*.*)*. Accidental events, during a night out, that may have been resolved when sober, were seen to facilitate fighting in males. “*At first they’ll be alright then as they get drunk and if somebody by accident pushes them they’ll get angry they might start a fight (G7; P3*, *F*, *15 yrs*.*)*.

Females were less prominent in participants’ accounts of alcohol-related aggression, and often mentioned to provide a contrast to males; “*I just don’t think the girls are as-as aggressive (G7; P2*, *F*, *15 yrs*.*)” the way they normally act as well affects them because like normally like boys are more into fights than girls” (G7; P5*, *F*, *14 yrs*.*)* or as non-participating bystanders. Participants suggested that if women did engage in aggressive behavior it was more likely to be expressed verbally: *“it’s more stereotypical for women to talk behind each other’s backs// it’s more argumental with women but with guys it’s more fight*, *fight*, *fight and everything” (G6; P4*, *M*, *15 yrs*.*)*.

Both male and female participants allocated women the roles of victims of male aggression. Interestingly, no participant offered the observation that males ought to reduce their potential for aggression by reducing their drinking, but several stated that females’ drinking conferred vulnerability; “*if they drink throughout the night they might become more vulnerable” (G2; P1*, *F*, *14 yrs*.*)*. This included getting robbed, unwanted pregnancy or being sexually assaulted. In several cases, participants suggested that women victimized by males may be too drunk; *“they* [females] *won’t be able to control themselves in a situation like that…*..*they might get a bit too drunk and then someone could take advantage of them” (G5; P4*, *M*,*15 yrs*.*)*.

### Alcohol-related aggression as an (almost) excusable action

When asked, participants did not see individuals engaging in alcohol-related aggression as blameworthy. As aggression was seen as neither a matter of choice nor agency, protagonists were excused because they did not intend to be aggressive; *‘well at the beginning of the night they’re just going out as usual just havin a bit of a drink and that but when they’re like getting a bit drunk kinda thing they don’t possibly mean to get into fights because you do silly things when you get drunk*, *being lads’ (G6*, *P4*, *M*, *15 yrs*.*)*. The ending to this statement ‘*being lads*’, connotes this relinquishment of responsibility that was typical of participants’ responses to questions about the moral status of alcohol-related aggression. Intention, rather than behavior, was held to be blameworthy. Thus, a consequence of participants’ beliefs of the inevitability of male alcohol-related aggression was that they did not feel males could be blamed for actions that were not personally controllable.

It could be argued that participants sought to ‘neutralise’ the issue of moral culpability to avoid responsibility for making judgments of others [28]. We do not believe this to be true, because participants allocated blame when they felt intention did exist. Several participants described a group of “*angry people” (G1; P3*, *F*, *16 yrs*.), whom they felt used intoxication as a cover or excuse for aggression. Comments showed that participants considered this group to be blameworthy because they intended to fight; *“There’s a group of guys and they don’t like another group of guys they might go to start a fight with them cos they know that’s the only way that they might get away with being in a fight*…*they do it when they’re drunk they can just say oh well I was drunk I didn’t even know them” (G6; P2*, *M*, *15 yrs*.*)*. “….. *like people who are just angry anyway … so when they’re drunk all that just kind of*, *comes together and they just get angry and start fights with people for like no particular reason really just because maybe they want the attention” (G1; P3*, *F*,*16 yrs*.*)*.

The behavior of this sub-group of males was seen to be less excusable because they used alcohol, not as a disinhibitor, but as a means to excuse their aggression; “*They might not even be drunk they might just hit them but it’s just a excuse to carry to get away with something” (G7; P3*, *F*, *15 yrs)*. *‘If he went the first time yeh that would maybe be an excuse but if it came to the point where you were continuously being violent it could be like hang on a minute this maybe isn’t the alcohol*… *you’re just using alcohol as an excuse like” (G5; P5*, *M*, *15 yrs*.*)*.*”*

### Scripts were informed by television

Few participants felt that their views of alcohol-related aggression were influenced by social media, videos, games or music. Instead, accounts focused on television, with particular reference to news and current affairs and reality programs. These influenced participants’ views on the frequency of alcohol-related aggression; “*the only thing you hear on the news about being drunk is when people start fights … they might just’ve turned 18 or something going round town and they might expect to be in a fight and they might just think that it’s they’ve not had a proper night out unless they’ve been in a fight so they might get in a fight just to feel like they’ve had a full clubbing experience” (G6; P6*, *M*, *15 yrs*.*)*. News, current affairs and reality television influenced participants’ views because participants felt that their portrayals were ‘*real*’; “*it has more of an effect when it’s on the news cos news is actually real live things so when it says that a woman’s been assaulted been raped*… *by another guy*…*that’s actually happened” (G6; P2*, *M*, *15 yrs*.*)*. The reality television programs that participants cited feature extravagant characters and extreme lifestyles, but participants remarked that the portrayals were real; *“TV programs like …its basically that people go on holiday go out clubbing with their friends*, *get really drunk*. *It shocks what happens on the nights outs” (G2; P1*, *F*, *14 yrs*.*)*.

Although participants generally described news, current affairs, and reality TV accounts of alcohol-related aggression as realistic, some wondered whether these depictions were entirely trustworthy. The quote from P6 of G6 in the previous paragraph began with the statement ‘*the only thing you hear on the news about being drunk is when people start fights’*, perhaps indicating an awareness that selective reporting may induce a distorted picture. This participant later stated *“I don’t know whether it’s the media representing them when you see on the news more women seem to be the victims than the guys do*… *see it a lot on television programmes like- like soaps’ (G6; P6*, *M*, *15 yrs*.*)*. Other members of G6 agreed that the media can also create a perception of reality: *“It’s like fog hiding the truth cos like the media’s like a big fog hiding what’s really happening it’s like they put this haze up that there’s always gonna be a fight … If you do watch that news you actually believe that this is what you have to do to be normal then those people will do that (G6; P3*, *M*, *15 yrs*.*)*. Nonetheless, the tendency to suggest that the media creates a distorted picture of reality was stronger for dramatic presentations, such as films and soap operas, than it was for news or current affairs.

## Discussion

Superficially, some aspects of participants’ accounts were similar to those of older drinkers in previous qualitative studies; they described a confluence of maleness, alcohol-related disinhibition, and random incidents that lead men to conflict. However, their accounts differed greatly from those of experienced drinkers, and, indeed, what is known about alcohol-related aggression. In particular, a number of participants saw alcohol-related aggression as genetically ‘*hardwired*’, and, thus, less morally compromised. Underpinning this was a narrow view of masculinity that focused almost exclusively on an exaggerated potential for aggression. This view precluded the understandings, expressed by drinkers in previous research, that a masculine role could encompass moral responsibility and self-control [8]. We do not suggest that holding such views will inevitably lead to alcohol-related aggression in later life. For example, we acknowledge that a confluence of social and developmental influences are likely to cause aggression amongst inexperienced drinkers. However, we do suggest that the accessibility and content of such scripts, if widely held, may help to explain why some inexperienced young people are pre-disposed to laboratory alcohol-related aggression [11].

First, participants across all groups were easily able to envisage male aggression, and were able to describe it in graphic, although exaggerated, detail. Script theory posits that the ease with which people can access scripts influences behavior, and is supported by laboratory studies showing that the availability of aggressive thoughts predicts alcohol-related aggression [29]. Moreover, descriptive norms are powerful drivers of alcohol-related aggression [30]. The perception that alcohol-related aggression is common and practiced by normal males may facilitate the likelihood that young males will engage in it [31].

Second, our participants spoke of a narrow and highly stereotyped view of masculinity. Whilst adult drinkers in previous research spoke of masculine expectations, values and behavior, they framed those in cultural terms [10, 7, 8]; our participants employed explicitly biological frameworks. Further, experienced drinkers see male aggressiveness and competitiveness as important aspects of masculinity, but report subtle temporal shifts in their ideas of masculine identity over their drinking careers—away from an unsophisticated belief that masculine identity is established through conflict, and toward a more nuanced sense of self-reliance that also prizes conflict avoidance and de-escalation. Our participants held a less nuanced view of masculinity. Lacking an appreciation of subtle expressions of masculinity, inexperienced drinkers may be more likely to see aggression as a viable response to perceived slight or challenge [10, 32]. It is notable that the experienced drinkers interviewed by Benson and Archer [10] attributed what they saw as a greater risk of alcohol-related aggression in younger males to a simpler sense of masculinity than theirs. Whilst we acknowledge that simple youthful impulsivity may lead to aggression amongst inexperienced drinkers, distorted views of masculinity may contribute to alcohol-related aggression.

Third, participants drew, or failed to draw, moral inferences from the alcohol-aggression scripts they held. Their biologically-based views meant that they saw alcohol-related aggression as beyond the control of perpetrators, and, thus, they did not take a moral view of it. Moral culpability was limited because aggression was not intended. Participants only voiced moral views when speaking of the ‘angry people’ whose intentions were aggressive. Again, participants’ views differ to adult findings. Benson and Archer [10], Tomsen [8], and Lindsay [7] describe a developmental narrowing of the parameters whereby alcohol-related aggression is regarded as reasonable. Aggression and self-defense may be seen as appropriate in older drinkers in some circumstances, but the development of a wider sense of moral and social norms act as counterweights to aggression [33].

Account should be taken of the developmental and ideological contexts in which participants acquired their views. Participants had little or no direct experience of late-night drinking. Nonetheless, they showed an awareness of cultures where drinking is frequently seen as stimulating and desirable, and where alcohol-related aggression is accepted and respected [10]. News, current affairs, and some reality programming influenced participants’ exaggerated perceptions of incidence and made alcohol-related aggression salient to them. Parallels to this finding have emerged in studies of media coverage of crime, terrorism, and infectious disease. Dramatic coverage can grossly exaggerate individuals’ perceptions of risk, simply because coverage makes instances of these more easily available to recall [34]. Thus, media coverage establishes a descriptive norm [35], offering a picture of what happens during late night drinking in a social environment that is otherwise largely unfamiliar to them.

Participants’ learning from media was active and skeptical. They did not directly assimilate editorial views, and one group went so far as to suggest that, to an extent, the media created rather than reflected reality. Our experience of many of the cited programs is that these provide moralistic condemnations of alcohol-related aggression. Conversely, with the exception of ‘angry people’, participants did not take a moral position. Instead, they imposed their own, explicitly biological, explanations onto what they had seen. In our experience, the media depictions that participants cited show some level of gender stereotyping, but these do not promote the biological determinism that we observed in participants.

Indeed, participants’ views of alcohol-related aggression appear to owe as much to a wider gender role stereotyping as they do to news or reality television. Aggression was seen as exclusively male, for which males were not held responsible. Females were seen as passive onlookers or victims, and sometimes responsible for their victimization by becoming intoxicated. These interpretations reflect gender research, pointing to childhood and adolescence as periods when sexist assumptions about gender are established and asserted as biological realities [36]. In particular, the attribution of traits such as competitiveness, dominance and aggression confers agency and power to males without moral responsibility. Similarly, female roles are characterized by helplessness and passivity. The employment of an explicitly biological framework provides a permanence and inevitability to these views [36]. Thus, alcohol-related aggression script content may be influenced by a wider pattern of stereotyping of young males as inherently aggressive and young women as potential victims of this aggression. We do not know how these views will influence how participants will view alcohol-related aggression when they reach drinking age, but they point to associations between extreme interpretations of masculine values and alcohol-related aggression [37].

This research allows insights into the origins, content, and possible behavioral implications of adolescents’ alcohol-related aggression scripts. It is tempting to also link our findings to expectancy research [38]. Alcohol expectancies seem to be socially learned in young people [39], but little is known about how alcohol-related aggression expectancies develop. Our findings show it is feasible that alcohol aggression expectancies are partly socially-learned. However, we counsel caution in extending our findings to expectancy theory. Expectancies are narrowly construed as individuals’ specific expectations of their individual behavior, whereas our findings address how young people generally interpret normative social behavior.

Interpretation of this research should be informed by conceptual and methodological limitations. Our claim that findings represent script learning rests upon participants’ lack of personal experience of either drinking or alcohol-related aggression. We did not attempt to establish whether participants had personal experience of drinking or had been aggressors, victims, or onlookers of alcohol-related aggression. Nonetheless, we cite previous findings that most participants of these ages do not drink, and that those who do drink are no more likely to engage in alcohol-related aggression than their peers who do not [11]. Similarly, by encouraging participants not to discuss personal experiences of alcohol-related aggression or those associated with their families, we may have missed key experiences that contributed to their views or contextual information that may allow us to understand the meanings of these views or why they were held.

## Conclusion

Our findings emphasize the importance of broad socio-cultural ideas and norms about alcohol-related aggression, specifically, and gender generally. These are probably socially learned. Young people develop scripts about alcohol-related aggression through televisual media, tempered by the pre-existing ideological frameworks through which they interpret their observations. Based on the success of general anti-violence interventions delivered by expert practitioners to adolescents in school and community settings [20, 40], we see particular value in programs that challenge views about the inevitability and legitimacy of male aggression. In particular, the idea that males are inherently aggressive can be challenged through a focus on more positive aspects of masculinity. Televisual media seems to have contributed to participants’ perceptions that alcohol-related aggression is a widespread consequence of male drinking. Further investigations can shed light on the role of media coverage on young peoples’ perceptions of the incidence of aggression after alcohol use, and these findings can be used to develop young people’s skills in taking a critical approach to what they learn from media that sensationalize alcohol-related aggression.
